# Hyperphosphatemia-Induced Oxidant/Antioxidant Imbalance Impairs Vascular Relaxation and Induces Inflammation and Fibrosis in Old Mice

**DOI:** 10.3390/antiox10081308

**Published:** 2021-08-19

**Authors:** Ana Asenjo-Bueno, Elena Alcalde-Estévez, Mariam El Assar, Gemma Olmos, Patricia Plaza, Patricia Sosa, Patricia Martínez-Miguel, María Piedad Ruiz-Torres, Susana López-Ongil

**Affiliations:** 1 Unidad de Investigación de la Fundación para la Investigación Biomédica del Hospital Universitario Príncipe de Asturias, Alcalá de Henares, 28805 Madrid, Spain; luaasbu19@gmail.com (A.A.-B.); patricia-4@hotmail.com (P.P.); pmmiguel@salud.madrid.org (P.M.-M.); 2 Departamento Biología de Sistemas, Universidad de Alcalá, Alcalá de Henares, 28871 Madrid, Spain; elena.alcaldee@edu.uah.es (E.A.-E.); gemma.olmos@uah.es (G.O.); patricia.sosacalle@gmail.com (P.S.); mpiedad.ruiz@uah.es (M.P.R.-T.); 3 Fundación para la Investigación Biomédica del Hospital Universitario de Getafe, Getafe, 28905 Madrid, Spain; mariam.assar@salud.madrid.org; 4 Instituto Reina Sofía de Investigación Nefrológica (IRSIN) de la Fundación Renal Iñigo Álvarez de Toledo (FRIAT), 28003 Madrid, Spain; 5 Area 3-Fisiología y Fisiopatología Renal y Vascular del IRYCIS, 28046 Madrid, Spain; 6 Servicio de Nefrología del Hospital Universitario Príncipe de Asturias, Alcalá de Henares, 28805 Madrid, Spain

**Keywords:** aging, hyperphosphatemia, endothelial dysfunction, fibrosis, reactive oxygen species, inflammation

## Abstract

Aging impairs vascular function, but the mechanisms involved are unknown. The aim of this study was to analyze whether aging-related hyperphosphatemia is implied in this effect by elucidating the role of oxidative stress. C57BL6 mice that were aged 5 months (young) and 24 months (old), receiving a standard (0.6%) or low-phosphate (0.2%) diet, were used. Isolated mesenteric arteries from old mice showed diminished endothelium-dependent vascular relaxation by the down-regulation of NOS3 expression, increased inflammation and increased fibrosis in isolated aortas, compared to those isolated from young mice. In parallel, increased Nox4 expression and reduced Nrf2, Sod2-Mn and Gpx1 were found in the aortas from old mice, resulting in oxidant/antioxidant imbalance. The low-phosphate diet improved vascular function and oxidant/antioxidant balance in old mice. Mechanisms were analyzed in endothelial (EC) and vascular smooth muscle cells (SMCs) treated with the phosphate donor ß-glycerophosphate (BGP). In EC, BGP increased Nox4 expression and ROS production, which reduced NOS3 expression via NFκB. BGP also increased inflammation in EC. In SMC, BGP increased Collagen I and fibronectin expression by priming ROS production and NFκB activity. In conclusion, hyperphosphatemia reduced endothelium-dependent vascular relaxation and increased inflammation and vascular fibrosis through an impairment of oxidant/antioxidant balance in old mice. A low-phosphate diet achieved improvements in the vascular function in old mice.

## 1. Introduction

The endothelium is a dynamic and functional organ involved in the regulation of many important biological functions, including maintenance of vascular tone and permeability, immunity, inflammatory responses, control of the coagulation process and angiogenesis promotion [1,2,3]. Vascular function is regulated by secreting molecules that act in an autocrine and paracrine manner, with nitric oxide (NO) being one of the most important mediators of endothelial function [4,5,6] apart from the redox balance [7] and endothelin-1 (ET-1).

Aging also modifies the arterial wall by changing the structure and function of vascular cells. In this way, SMCs tend to modify their phenotype from contractile to synthetic, determining the media layer thickness [8]. These structural changes are associated with an increase in collagen [9] and a reduction in elastin content [10], reversing the elastin/collagen ratio [11,12,13,14,15] and increasing the vessel stiffness. In line with this, arterial stiffness [16,17], considered a key feature of aging-related vascular alterations, is always preceded by an impaired endothelial vasodilation suggesting that it is also linked to endothelial dysfunction. Tissue fibrosis is characterized by the excessive deposition of ECM proteins, such as collagens and fibronectin (FN) [18] in different organs; playing a critical role in the detrimental damage of these organs [19]. Fibrosis is triggered by the action of growth factors such as TGF-ß1 and connective tissue growth factor (CTGF or CCN2), resulting in fibroblast activation or inflammation processes [19,20]. In fact, the appearance of fibrotic areas in the aorta could be involved in the development of endothelial dysfunction. A typical early feature of vascular aging is a deterioration of endothelial vasodilatation that precedes the clinical manifestations of endothelial dysfunction.

Nowadays, vascular aging is considered a modifiable risk factor [21]. Accordingly, the preservation of endothelial function and vessel wall structure is fundamental for healthy aging [21,22].

Factors involved in vascular aging are beginning to be understood. Hyperphosphatemia is a pathological condition also related to aging and chronic kidney disease (CKD) [23,24,25]. Phosphate excess is considered as a critical factor in the pathogenesis of mineral and bone disorders associated with CKD and recently determined to have toxic effects on the cardiovascular system and in the aging process [26]. Mice deficient in Klotho or fibroblast growth factor 23 (FGF23) manifest a premature aging syndrome associated with hyperphosphatemia, which can be rescued by reducing blood phosphate levels with dietary interventions [23]. Phosphate also impairs endothelial function [27,28]. High phosphate levels increase oxidative stress and suppress the bioavailability of NO through inhibition of phosphorylation that activates NOS3 [27,29]. A dietary phosphate restriction not only reduced hyperphosphatemia but also improved the impaired vasodilation of the aorta in adenine-induced kidney disease rats [30].

Oxidative stress is induced by the imbalance between oxidants production and antioxidant capacity of the cells and is involved in many diseases, such as cancer and inflammation, and it plays a relevant role in aging [31,32,33,34]. It is also well known that oxidative stress can modify endothelial function [21,35]. Several studies have demonstrated that oxidative stress and inflammation are the most important pathological processes that determine endothelial dysfunction in healthy older adults and in rodent models [36,37,38]. In aging, a rise in blood glucose, obesity, cholesterol, blood pressure and sodium intake can regulate endothelial dysfunction through oxidative stress and inflammation [39,40,41,42,43,44].

Moreover, proinflammatory cytokines, such as tumor necrosis factor alpha (TNF-α), interleukin 6 (IL-6) and monocyte chemoattractant protein-1 (MCP-1) [45] increase with age in healthy individuals [45,46]. Their upregulation in aging produces a chronic low grade inflammation termed inflammaging, which is associated with aged-diseases [47].

Aging [1,11,12] and hyperphosphatemia impair endothelial function [27] by unknown mechanisms. Although a phosphate restriction diet can rescue the impaired endothelial function in animal models associated with kidney disease [30], there are still no studies in aging animal models. Thus, the aim of the present work was to analyze whether hyperphosphatemia was involved in aging-related vascular dysfunction, characterizing the mechanism involved in this effect.

## 2. Materials and Methods

### 2.1. Materials

Culture plates, culture media, BlueStar-prestained protein marker, BCA protein assay reagent, nitrocellulose membrane, secondary horseradish peroxidase-conjugated goat anti-mouse IgG and CL-Xposure films were from Cultek (Thermo Fisher Scientific, Madrid, Spain); Supersignal West Pico detection system and LightShift Chemiluminescent EMSA kit were from Pierce (Thermo Fisher Scientific, Madrid, Spain). CellROX deep red probe for oxidative stress detection were from Molecular Probes; dihydroethidium (DHE) probe, NucBlue live cells stain Ready probe reagent and Prolong Gold antifade reagent were purchased from Invitrogen (Thermo Fisher Scientific, Madrid, Spain). Rabbit polyclonal anti-Fibronectin antibody (ab6584), rabbit polyclonal anti-Collagen-I antibody (ab34710), rabbit polyclonal anti-Collagen V (ab7046), rabbit polyclonal anti-CTGF antibody (ab6992), mouse polyclonal anti-Fibronectin antibody (ab6328), Elastic Connective Tissue Stain kit (ab150667) and Picro Sirius Red Stain kit (ab150681) were from Abcam (Cambridge, UK). Acrylamide-bisacrylamide was from Hispanlab-Pronadisa (Madrid, Spain). Trizol reagent and RNA later solution were from Ambion-Life technologies (Thermo Fisher Scientific, Madrid, Spain). Mouse monoclonal anti-nitrotyrosine (sc-32757) and anti-^Ser32^ P-IκBα (sc-8404) antibodies were from Santa Cruz Biotechnology (Heidelberg, Germany). Rabbit polyclonal anti-^Ser536^ P-NFκB-p65 antibody (3033S) was from Cell Signaling Technology (Werfen, Barcelona, Spain). Rabbit polyclonal anti-Nox4 antibody (GTX21929) was from GeneTex (Labclinics, Barcelona, Spain). Mouse ^Ser1177^ P-NOS3 (612393) and mouse NOS3 (610297) antibodies were from BD Transduction Laboratories (BD BioSciences, Bath, United Kingdon). Protease inhibitor cocktail tablets and FastStart universal probe master were from Roche Diagnostics S.L. (Barcelona, Spain). High-capacity cDNA reverse transcription kit and TaqMan gene expression assays from mice were purchased from Applied Biosystems (Thermo Fisher Scientific, Madrid, Spain). Antagonists from endothelin receptor type A, BQ123, mouse polyclonal anti-GAPDH antibody (G8795), ß-glycerophosphate (G9891), acetylcholine chloride (A9101), sodium nitroprusside (S0501), parthenolide (P0667), aspirin (acetyl salicylic acid, PHR1003) and norepinephrine (N5785) were purchased from Sigma-Aldrich-Fluka Chemical Co. (St. Louis, MO, USA).

### 2.2. Cell Culture

For in vitro studies, we used human endothelial cells (EC, EA.hy926) from ATCC and human SMC from a primary culture, donated by Universidad Autónoma de Madrid. ECs were grown in Dulbecco’s Modified Eagle Medium (DMEM) from ATCC (30-2002) and SMC in DMEM supplemented with 4 mmol/L L-glutamine from Sigma (D6171), both containing 4.5 g/L glucose and supplemented with 10% fetal bovine serum, 100 U/mL penicillin and 100 µg/mL streptomycin, in an atmosphere of 95% air and 5% CO_2_.

### 2.3. Experimental Designs

In order to evaluate the in vitro effect of hyperphosphatemia, we used the phosphate donor ß-glycerophosphate (BGP) to treat human EC and SMC at different times with 10 mM BGP, a dose chosen based on previous studies [28]. The cells were grown to confluence, and then they were incubated in serum-free medium for 24 h before adding treatment. ROS production was inhibited using the antioxidant N-acetyl-cysteine (NAC, 100 µM).

### 2.4. Animal Studies

For in vivo studies, we used male C57BL6 mice from 5 to 24 months of age obtained from Janvier Laboratories. All mice were kept on a 12:12 h light-dark cycle, at 24 °C and food and water were available ad libitum. Twenty-four month old mice were divided into two groups: one of them received a low P diet containing 0.2% P, 0.9% Ca and 0.2% Mg (Experimental diet: S9723-E022 from SSNIFF Spezialdiäten GmbH) for the last 12 weeks of their life, whereas the other group continued receiving the standard diet containing 0.6% P as the group of young mice did. Before sacrifice, animals were anesthetized with isofluorane, and blood samples were collected by heart puncture exsanguinations. The aortas were isolated and conserved in RNA later solution for protein or RNA extraction or collected in p-formaldehyde for histological studies. Omentum (for isolation of mesenteric small vessels) was carefully excised for the functional evaluations. Serum phosphate was measured by QuantiChrom phosphate assay kit (DIPI-500) from Bioassay Systems (Deltaclon SL, Madrid, Spain) using a microplate reader. General data of mice including body weight, body mass index (BMI), food intake and water intake are shown in the Supplementary Figure S1A.

### 2.5. Vascular Reactivity of Mice Mesenteric Arteries

First to second order branches of the mesenteric arterial tree were obtained from omentum specimens and dissected by carefully removing the adhering fat tissue. Arterial ring segments (~2 mm long and 238 ± 6.1 µm diameter in average) were subsequently mounted on small-vessel wire myographs (Danish MyoTechnology, Aarhus, Denmark) for circular isometric tension recordings, as previously described [48,49,50]. The vessels were allowed to equilibrate for 30 min in Krebs–Henseleit solution (KHS) continuously bubbled with a 95% O_2_/5% CO_2_ mixture to maintain a pH of 7.4. The passive tension and internal circumference of vascular segments when relaxed in situ under a transmural pressure of 100 mmHg (L100) were determined. The arteries were then set to an internal circumference equivalent to 90% of L100, at which the force development is close to maximal. In order to assess vessel viability, preparations were then exposed to 125 mM K+ (KKHS, equimolar substitution of NaCl for KCl in KHS), and the contractile response was measured. After a washout and stabilization period, mice mesenteric arteries were contracted with 1–3 µM norepinephrine (NE, 80% of KKHS-induced contraction, approximately) and relaxation responses were evaluated by cumulative additions of acetylcholine (ACh, 1 nM to 10 µM) or sodium nitroprusside (SNP, 1 nM to 10 µM) to the chambers. In some experiments, a non-specific NOS inhibitor, L-nitro-arginine methyl ester (L-NAME, 100 µM), was added 30 min before the concentration-response curves to ACh were started.

### 2.6. Sirius Red and Elastin Staining and DHE Detection

Aortas, heart and kidneys were collected in 4% p-formaldehyde and then processed in paraffin. Afterwards, samples were de-paraffined in xylene and then hydrated in descending order of ethanol dilutions to finally be stained with the Picro Sirius Red Stain kit to assess fibrosis or with the Elastic Connective Tissue Stain kit to visualize elastin fibers. Kits were used according to the manufacturer’s instructions for 30 min to visualize collagen fibers in red and muscle fibers in yellow with the Picro Sirius Red solution or for 15 min to visualize elastin in black with the Elastic Stain solution. After that, they were dehydrated and mounted with DPX solution to be observed with a microscope. Pictures were obtained with 40× magnification, the intensities of Sirius red or black elastin were measured by using Image Pro Plus software (www.mediacy.com/imageproplus). In addition, aorta thickness was measured in those pictures using Image J software (http://rsbweb.nih.gov/ij/).

A DHE probe was used to detect reactive oxygen species in aorta tissue, which exhibits blue-fluorescence in the cytosol until oxidized, where it intercalates within the cell’s DNA, staining its nucleus a bright fluorescent red. Samples were de-paraffined as described above, and antigen retrieval was performed by heat mediation in a citrate buffer pH 6. After that, the samples were incubated 30 min at 37 °C with 4 µM DHE, washed with PBS and finally treated with DAPI to stain nuclei (NucBlue live cells stain Ready probes reagent from Invitrogen) for 20 min at R/T. Lastly, slides were mounted with the reagent Prolong Gold antifade from Invitrogen. The slides were analyzed with a LEICA TCS-SP5 confocal microscope (Leica Microsystems; GmbH, Mannheim, Germany) using the helium-neon laser at 633 nm to detect red fluorescence of DHE probe and at 405 nm to detect blue fluorescence of nuclei stained with DAPI. Pictures were obtained with 40× magnification and fluorescence intensity of DHE, and number of nuclei were quantified using Image J software (http://rsbweb.nih.gov/ij/).

### 2.7. Immunohistochemistry of Nitrotyrosine and Nox4

Aorta slices were de-paraffined as described above, and antigen retrieval was by heat mediation in a citrate buffer pH 6. Samples were blocked with 10% goat serum and 1% BSA in PBS for 1 h at R/T. After that, they were permeabilized for 10 min with 0.2% Triton X-100, and after 3 washes they were incubated with mouse anti-Nitrotyrosine (1:10 dilution) and rabbit anti-Nox4 (1:50 dilution) antibodies with 3% BSA in PBS at 4 °C overnight. After 3 washes, endogenous peroxidase was blocking with 3% H_2_O_2_ for 15 min at R/T. The samples were incubated with the secondary antibody at 1:100 dilution, anti-mouse for Nitrotyrosine and anti-rabbit for Nox4 with 3% BSA in PBS for 1 h at R/T. Then, samples were washed and brown color was developed using DAB substrate kit. After that, they were dehydrated and mounted with DPX solution to be observed with a microscope. Pictures were obtained with 40× magnification, and the intensity of the brown color were measured using Image Pro Plus software (www.mediacy.com/imageproplus).

### 2.8. Quantitative RT-PCR

Total RNA from endothelial cells or aorta tissue from mice were isolated using Trizol reagent according to the manufacturer’s protocol. cDNA was synthesized using a High Capacity cDNA reverse transcription kit [28,51]. The expressions of IL-1 beta, IL-6, TNF-alfa and MCP-1 in endothelial cells and the expression of endothelial nitric oxide synthase (Nos3), inducible nitric oxide synthase (Nos2), Nrf-2 factor (Nfe2l2), glutathione peroxidase-1 (Gpx1) and superoxide dismutase-2 Mn (Sod2-Mn) in aortas from mice were determined by quantitative PCR (ABI Prism 7500 Fast Real-Time PCR System) and analyzed with 7500 Fast sequence detection software v1.3.1 (Applied Biosystems Inc., Foster City, CA, USA), using specific TaqMan assays and Double delta Ct method. TaqMan probes used for mice were Nos3 (Mm00435217_m1), Nos2 (Mm00440502_m1), Nfe2l2 (Mm00477784_m1), Gpx1 (Mm00656767_g1), Sod2-Mn (Mm01313000_m1) and Actb (Mm01205647_g1), and for human cells they were IL1B (Hs01555410_m1), IL6 (Hs00174131_m1), CCL2 (Hs00234140_m1), TNF-alfa (Hs00174128_m1) and ACTB (Hs99999903_m1).

### 2.9. Western Blot Assays

Proteins were extracted from cells or aortas by using the Lysis Buffer (20 mM Tris-HCl pH 7.5, 1 mM EGTA, 1 mM EDTA, 150 mM NaCl, 10 mM sodium pyrophosphate, 1% Triton X-100 and 0.1% sodium deoxycholate) containing a protease inhibitor cocktail. Lysis solution was spun at 13,000 rpm for 30 min at 4 °C. Protein concentration was determined with a protein assay kit from BioRad. Samples (30 µg protein/lane) were run on SDS-polyacrylamide gels (PAGE) under reducing conditions and transferred onto nitrocellulose membranes, with the exception of gels made for the study of Collagen, which were made under non-reducing conditions. Membranes were blocked with 5% (*w*/*v*) non-fat dry milk for 1 h at room temperature (R/T) in Tween Tris buffered saline (TTBS) (20 mM Tris-HCl pH 7.5, 0.9% NaCl, 0.05% Tween 20). After that, they were incubated for 1 h at R/T with different specific antibodies for the detection of NOS3 (1:2500, 3% BSA), ^Ser1177^ P-NOS3 (1:2500, 3% BSA), Nox4 (1:2000, 0.05% BSA), FN (1:1000, 0.05% BSA), Collagen I (1:500, 3% BSA), Collagen V (1:2000, 1.5% BSA), CTGF (1:1500, 1.5% BSA), ^Ser32^ P-IκBα (1:1000, 3% BSA) and ^Ser536^ P-NFκB-p65 (1:1000, 3% BSA). After washing in TTBS, membranes were incubated for 1 h at R/T with secondary antibody, horseradish peroxidase-conjugated goat anti-mouse IgG (50,000-fold diluted for NOS3, ^Ser1177^ P-NOS3, and ^Ser32^ P-IκBα) or goat anti-rabbit IgG (10,000-fold diluted for Nox4, ^Ser536^ P-NFκB-p65, FN, Collagen I/V and CTGF). The immunoreactive bands were visualized with the SuperSignal West Pico detection system after 30–60 s of exposure to CL-Xposure films. Finally, membranes were re-incubated with a mouse anti-GAPDH antibody in order to normalize each protein level.

### 2.10. Immunofluorescence

SMCs were grown on cover slips and then treated with 10 mM BGP for 6 h in the presence or the absence of the antioxidant N-Acetyl-cysteine (NAC 100 µM) or the antagonist of phosphate receptor Pit-1 (PFA 1 mM). After being washed twice, cells were fixed with 4% p-formaldehyde for 10 min at R/T, and then they were incubated with 0.5% Triton X-100 for 10 min at R/T. Later, cells were blocked with 5% BSA for 1 h at R/T and incubated overnight at 4 °C with a mix of mouse anti-FN (1:200 dilution) and rabbit anti-Col-I (1:200 dilution) antibodies or with rabbit anti-CTGF (1:200 dilution) antibody. After being washed in PBS, cells were incubated with a mix of two secondary antibodies at 1:200 dilution, goat anti-mouse IgG labeled with Alexa Fluor 647 and goat anti-rabbit IgG labeled with Alexa Fluor 488. After that, cells were washed with PBS and incubated with DAPI to stain nuclei (NucBlue live cells stain Ready probes reagent from Invitrogen) for 20 min at R/T, 2 drops per milliliter. Lastly, the cover slips were mounted with the reagent Prolong Gold antifade from Invitrogen. Samples were analyzed using a LEICA TCS-SP5 confocal microscope (Leica Microsystems; GmbH, Mannheim, Germany) with helium-neon laser to detect red fluorescence at 633 nm for FN antibody labeled with Alexa Fluor 647 or with argon laser to detect green fluorescence at 488 nm for Col I antibody labeled with Alexa Fluor 488 and to detect blue fluorescence of nuclei stained with DAPI at 405 nm. Images were obtained, and fluorescence intensity was measured by densitometry by using Image J software (http://rsbweb.nih.gov/ij/).

### 2.11. Electrophoretic Mobility Shifts Assays (EMSA)

Nuclear extracts isolated from SMCs were assessed by electrophoretic mobility shift [28] to check on the activation of nuclear factor-kappa B (NFκB). DNA–protein interactions were detected with a nonisotopic method using the LightShift Chemiluminescent EMSA Kit. Oligonucleotide sequences were based on the putative NFκB binding element in the human FN promoter (from nucleotides 25 to 55; 5′-GGG GGA GGA GAG GGA ACC CCA GGC GCG AGC-3′) [52]. Biotin end-labeled DNAs containing the binding site of interest (NFκB from FN, biotin-NFκB) were incubated with 1 µg/µL nuclear extracts for 20 min at R/T. Protein–DNA complexes were subjected to gel electrophoresis on a native polyacrylamide gel in 0.5 x Tris Buffer EDTA and then transferred to a positively charged nylon membrane. The biotin end-labeled DNA was detected using the Streptavidin-Horseradish Peroxidase Conjugate and the Chemiluminescent Substrate as described in the kit. For competition experiments, 200-fold molar excess of competitor DNA (AP-1 oligonucleotides) was coincubated with biotin end-labeled DNAs (biotin-NFκB).

### 2.12. ROS Production

SMCs or ECs were grown in 60 µ-dishes 35 mm high with glass bottom (Ibidi, Martinsried, Munich, Germany) until 80% confluent and then incubated for 24 h with serum-free DMEM and finally treated with 10 mM BGP at different times. ROS production was measured by fluorescence confocal microscopy by using the CellROX Deep Red probe, with 5 µM CellROX being added and incubated for the last 30 min. At the end of incubation, the cells were washed twice with PBS and fixed with 4% p-formaldehyde for 15 min. Cells were analyzed using a LEICA TCS-SP5 confocal microscope (Leica Microsystems; Wetzlar, Germany) with helium-neon laser at 633 nm to detect red fluorescence of the CellROX probe. Pictures were taken, and the intensity of red fluorescence was measured by densitometry using the Image J software (http://rsbweb.nih.gov/ij/).

### 2.13. Statistical Analysis

GraphPad Prism 5 Software was used for statistical analysis. The following statistical tests were applied in cell experiments: one-way ANOVA followed by Dunnett’s post-tests for experiments compared with control cells or followed by Bonferroni post-tests for multiple comparisons. Statistical tests applied in experiments performed on animals were one-way ANOVA followed by Bonferroni post-tests for multiple comparisons, or two-way ANOVA in the case of the vascular reactivity assays for comparison of complete dose–response curves followed by Bonferroni post-test for multiple comparisons. Correlations were analyzed using the Pearson correlation non-parametric test. Unless otherwise specified, data are expressed as the mean ± standard error and expressed as a percentage of the control values of a variable number of experiments detailed in figure legends. The level of statistical significance was defined as *p* < 0.05.

## 3. Results

### 3.1. Hyperphosphatemia Induced Vascular Dysfunction in Old Mice by Reducing Endothelium-Dependent Vascular Relaxation and Increasing Inflammation and Fibrosis

**Figure 1:**
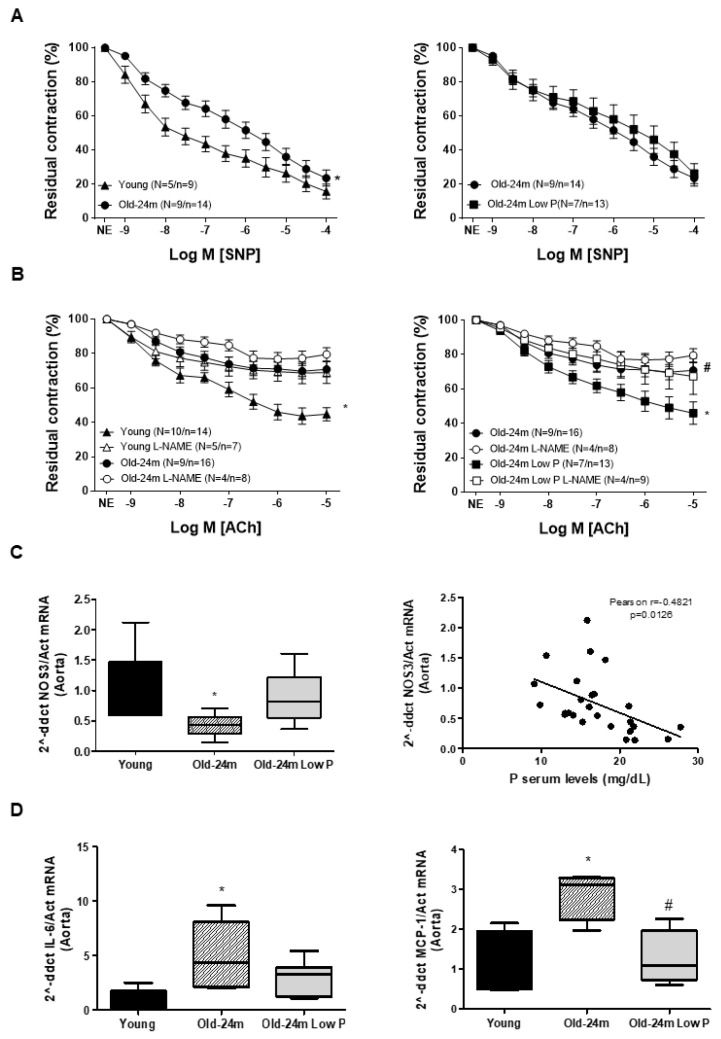
Hyperphosphatemia reduces endothelium-dependent vascular relaxation in old mice by reducing NOS3 expression and increases vascular inflammation. Male C57Bl6 mice from 5 month old (Young, closed triangle), 24 month old fed with normal diet (Old-24 m, closed circle) and 24 month old mice fed with a low phosphate diet for the last 3 months of life (Old-24 m Low P, closed square) were used. Vascular reactivity was measured in mesenteric arteries from those mice (**A**,**B**). Vascular relaxation in response to SNP are shown in panel A, comparing Young with Old mice on the left panel A and comparing Old-24 m with Old-24 m low P mice on the right panel A. N indicates the number of animals measured, and n denotes the number of segments analyzed. (**B**) Vascular relaxation in response to ACh was measured in the presence of inhibitor of NOS3, L-NAME 100 µM (open symbols) or in the absence of L-NAME (closed symbols). Young versus Old mice on the left panel B are compared with or without L-NAME treatment, and old mice fed with standard diet on the right panel B are compared with respect to old mice fed with a low P diet, with or without L-NAME. (**C**) On the left panel, NOS3 expression by real-time PCR of the aorta tissue from those mice is shown, and on the right the graph of correlation between P serum levels and NOS3 expression in aorta is shown (Pearson r = −0.6034, *p* = 0.0018). (**D**) IL-6 and MCP-1 expressions were analyzed in aorta by real-time PCR. Values are the mean ± SEM of 10 mice per group, * *p* < 0.05 vs. the other groups, # *p* < 0.05 compared Old-24 m vs. Old-24 m Low P.

We analyzed whether aging-related hyperphosphatemia was associated to age-related changes in vascular relaxation. For this purpose, we evaluated vascular reactivity in mesenteric artery rings isolated from young and old mice. Additionally, a 21 month old mice group received a low P diet for the last 3 months of life to reduce the phosphate intake in order to evaluate the role of hyperphosphatemia in vascular relaxation. Firstly, it was confirmed that our aging animal model from 24 months old mice presented hyperphosphatemia and that this could be reduced significantly with a restriction in phosphate intake. Phosphate levels in mg/dL (mean ± SEM) were as follows: Young mice, 13.55 ± 0.98; Old-24 m, 21.60 * ± 0.95; Old-24 m Low P, 16.67 ^#^ ± 0.84; * *p* < 0.05 Old-24 m vs. Young and ^#^ *p* < 0.05 Old-24 m Low P vs. Old-24 m. In addition to hyperphosphatemia, old mice exhibited a significant reduction in SNP and ACh-induced responses, endothelial independent and dependent vascular relaxation, respectively, compared to young mice (Figure 1A,B, on the left). Old mice fed with the low P diet showed an improvement of endothelium-dependent vascular relaxation in response to ACh (Figure 1B, on the right). By contrast, no changes were detected in the endothelium-independent vascular relaxation induced by SNP (Figure 1A, on the right). Additional experiments of vascular reactivity were performed in the presence of L-NAME, a NOS3 inhibitor, to confirm the potential role of NOS3 in the endothelium-dependent relaxation induced by ACh. When mesenteric arteries were exposed to L-NAME, relaxation in response to ACh became worse in all groups of mice (Figure 1B): young mice, old mice and even old mice fed with the low P diet. For that reason, it was checked whether NOS3 expression was reduced in old mice. The mRNA expression of NOS3 was analyzed in aorta by real time PCR, and it was diminished in old mice with respect to young mice, but it was improved in mice fed with the low P diet (Figure 1C). A negative statistical correlation was found between NOS3 expression and serum phosphate levels from those mice (Figure 1C).

In order to assess the effect of hyperphosphatemia on vascular inflammation, the pro-inflammatory cytokine IL-6 and monocyte chemoattractant protein-1 (MCP-1) were analyzed in the aortas from mice by real time PCR. Both increased significantly in aortas from old mice with respect to young mice, whereas mice fed with low P diet presented lower expression (Figure 1D). A significative correlation was found between IL-6 expression and P serum levels (Pearson r = 0.6789, *p* < 0.0002) (Supplementary Figure S1B).

**Figure 2:**
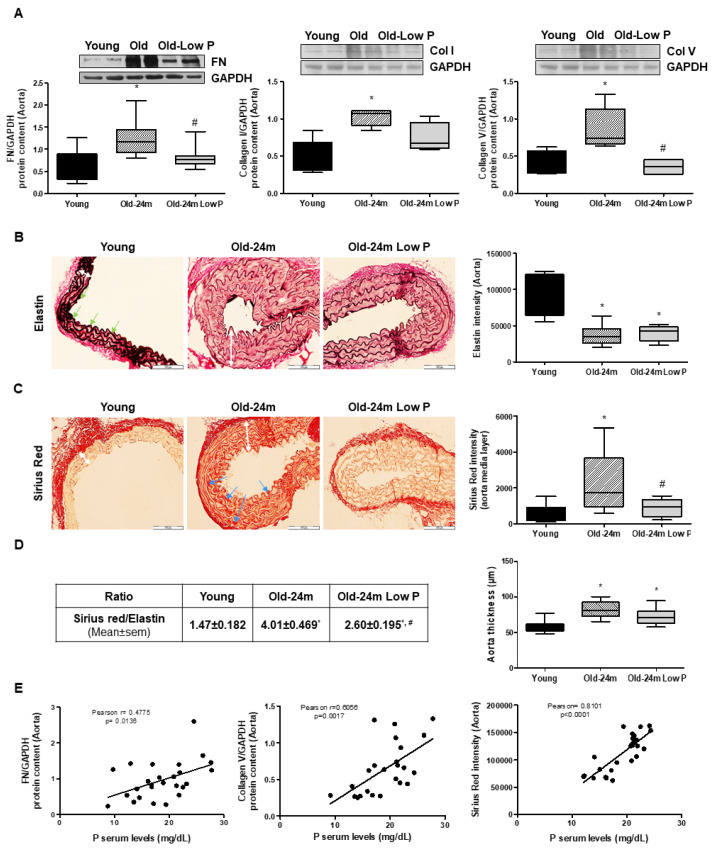
Hyperphosphatemia induces fibrosis in aorta from old mice. Male C57Bl6 mice from 5 month old (Young, closed bars), 24 month old fed with normal diet (Old-24 m, striped bars) and 24 month old mice fed with a low phosphate diet for the last 3 months (Old-24 m Low P, grey bars) were used. (**A**) Aorta tissue was isolated from mice to evaluate fibronectin (FN), collagen I (Col I) and collagen V (Col V) protein expressions by Western blot. A representative Western blot is shown above with the densitometric analysis below. (**B**,**C**) Elastin staining (**B**) and Sirius red staining (**C**) of the aorta from mice (40×) is shown on the left of each panel with the graph of densitometric analysis on the right. Scale bar, 100 µm. Green arrows indicate elastin expression in black, blue arrows indicate Sirius red expression in red and white arrows indicate the thickness of the aorta wall. (**D**) On the left, a table with the ratio between Sirius red and Elastin expression of each group of mice is shown, and a graph with the densitometric analysis of aorta thickness from all groups is shown on the right of the panel. (**E**) Graphs of correlations between P serum levels and different parameters of fibrosis are shown: P levels and FN expression (Pearson r = 0.4775, *p* = 0.0136), P levels and Col V expression (Pearson r = 0.6056, *p* = 0.0017) and P levels and Sirius red expression (Pearson r = 0.8101, *p* = 0.0001). Values are the mean ± SEM of 10 mice per group, * *p* < 0.05 vs. young, # *p* < 0.05 vs. Old-24 m.

Finally, we checked whether hyperphosphatemia was associated with aging-related vascular fibrosis. We compared the expression of ECM proteins in aortas isolated from young and old mice, with or without phosphate dietary restriction. Figure 2 illustrates a significant increase in the expression of FN, collagen I and collagen V (Figure 2A) analyzed by Western blot. Restriction in phosphate intake reduced the expression of these proteins (Figure 2A) with respect to old mice receiving a standard diet. The aorta wall from old mice had less elastin expression, assessed by immunohistochemistry (Figure 2B), and more appearance of fibrosis mainly located into the media layer of SMC, which was measured in media layer using Sirius Red staining (Figure 2C). The low P diet was able to significantly reduce the fibrosis from old mice, however, elastin expression did not change significantly. Since the blood vessels are divided depending on function, location and size, we have also included histological data from vessel wall changes from the other organs of these mice, such as the heart and kidney (Supplemental Figure S1D). Fibrosis was also detected in small vessels from heart and kidney samples from old mice, and it was reduced with the low P diet. Moreover, the ratio of Sirius red/elastin is significantly inverted between old and young mice (see table of Figure 2D, left panel) and was improved in mice fed with the low P diet. Even the aorta thickness was significantly increased in old mice, but a few changes after treatment with low P diet were detected (Figure 2D, right panel). We also identified a positive correlation between serum phosphate levels and the expression of FN, collagen V and Sirius red staining (Figure 2E). We even found a positive correlation between IL-6 expression and FN expression (Pearson r = 0.5813, *p* < 0.0036) (Supplementary Figure S1C), suggesting a potential association between fibrosis and inflammation.

### 3.2. Hyperphosphatemia Impairs Oxidant/Antioxidant Balance and Induces Nitrosative Damage in Aorta from Old Mice

**Figure 3:**
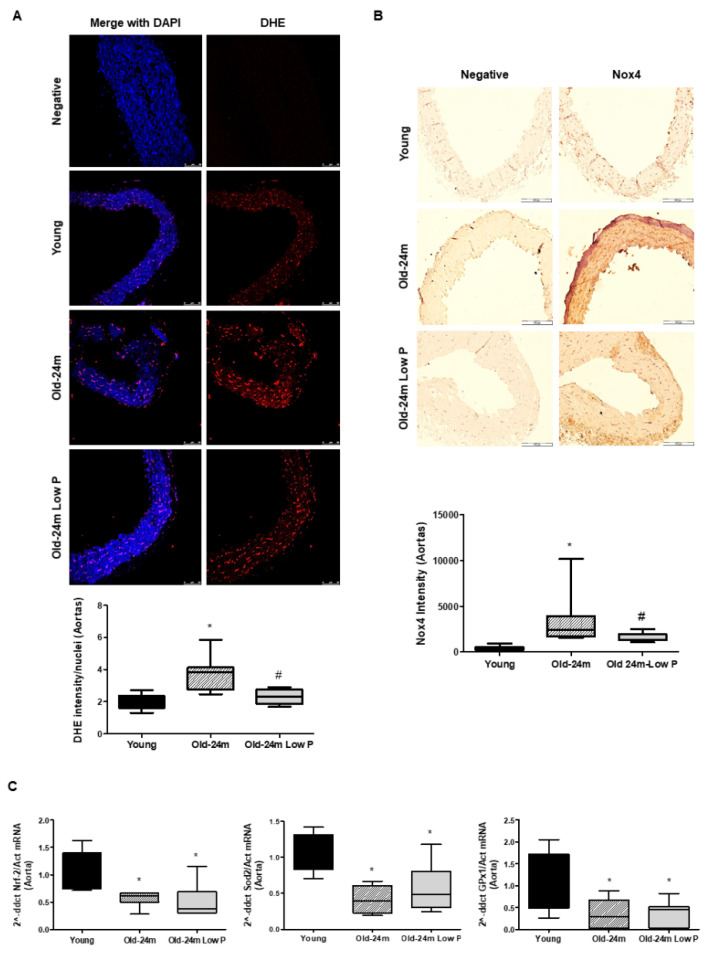
Hyperphosphatemia impairs oxidant/antioxidant balance in the aorta from old mice. Male C57Bl6 mice from 5 month old (Young, closed bars), 24 month old fed with normal diet (Old-24 m, striped bars) and 24 month old mice fed with a low phosphate diet for the last 3 months (Old-24 m Low P, grey bars) were used. (**A**) Oxidative stress in aorta tissue was detected with the DHE probe (in red fluorescence) by using a confocal microscope. Representative microphotographs are shown with 40× magnification and the densitometric analysis is shown below the pictures, where the bar graph represents the ratio between DHE intensity fluorescence and the number of nuclei. Scale bar, 50 µm. (**B**) Nox4 expression of the aorta from mice (40×) is shown with the graph of densitometric analysis below the pictures. Scale bar, 100 µm. (**C**) Antioxidant barrier was evaluated by the expression of Nrf-2 factor and antioxidants genes Sod2-Mn and Gpx1 analyzed by real time PCR in aortic tissue. Values are the mean ± SEM of 6 mice per group, * *p* < 0.05 vs. young, # *p* < 0.05 vs. Old-24 m.

In order to explore the mechanisms involved in the observed effects, we assessed whether hyperphosphatemia was related to oxidative stress. Old mice showed higher levels of ROS production, measured by immunofluorescence with the DHE probe, with respect to young mice (Figure 3A). Low P diet was able to significantly reduce ROS production from old mice (Figure 3A). As NADPH oxidase is an important source of ROS production, Nox4 expression was also measured in aorta. Old mice showed a significant increase in Nox4 expression, which was blocked with the low P diet (Figure 3B), suggesting that aging-related hyperphosphatemia could mediate ROS production through Nox4 activation. Moreover, the antioxidant barrier was also studied in aorta samples by qPCR using specific TaqMan probes for Nrf2 factor and the antioxidant enzymes Sod2-Mn and Gpx1 that are regulated by Nrf2. Nrf2 and antioxidant enzymes were reduced in old mice (Figure 3C). However, no changes were observed in old mice treated with low P diet. These results suggest an imbalance between oxidant production and antioxidant capacity in aorta from old mice in favor of ROS production.

**Figure 4:**
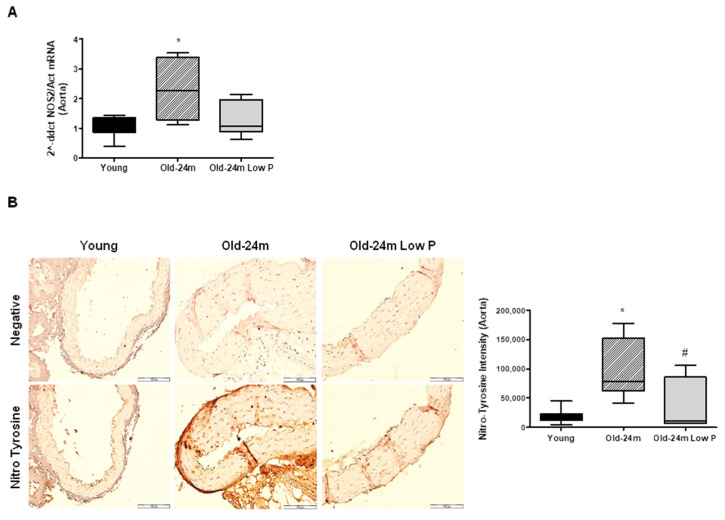
Hyperphosphatemia induces NOS2 expression and nitrosative damage in aorta from old mice. Male C57Bl6 mice from 5 month old (Young, closed bars), 24 month old fed with normal diet (Old-24 m, striped bars) and 24 month old mice fed with a low phosphate diet for the last 3 months (Old-24 m Low P, grey bars) were used. (**A**) NOS2 expression was evaluated by real time PCR in aortic tissue. (**B**) Nitrotyrosine expression is shown in the aorta slices from mice (40×) with the graph of densitometric analysis on the right panel. Scale bar, 100 µm. Values are the mean ± SEM of 6 mice per group, * *p* < 0.05 vs. young, # *p* < 0.05 vs. Old-24 m.

On the other hand, we studied whether hyperphosphatemia was related to aging-related nitrosative damage. For this purpose, NOS2 was analyzed in aorta from mice by real time PCR. NOS2 increased significantly in aorta from old mice with respect to young mice, whereas mice fed with low P diet blocked NOS2 induction (Figure 4A). Lastly, as NOS2 stress and oxidative stress were increased in old mice, nitrosative damage were assessed in aorta slices by Nitrotyrosine expression, finding a significant rise in old mice compared with young mice, which was significant reduced with the low P diet (Figure 4B).

These in vivo results from mice point to a potential relationship between aging-related hyperphosphatemia and the presence of vascular fibrosis, inflammation and oxidative stress in the aorta from old mice, which altogether could mediate endothelial dysfunction.

### 3.3. Hyperphosphatemia Downregulates NOS3 by Increasing Oxidative Stress through NFkB Activation in Endothelial Cells

**Figure 5:**
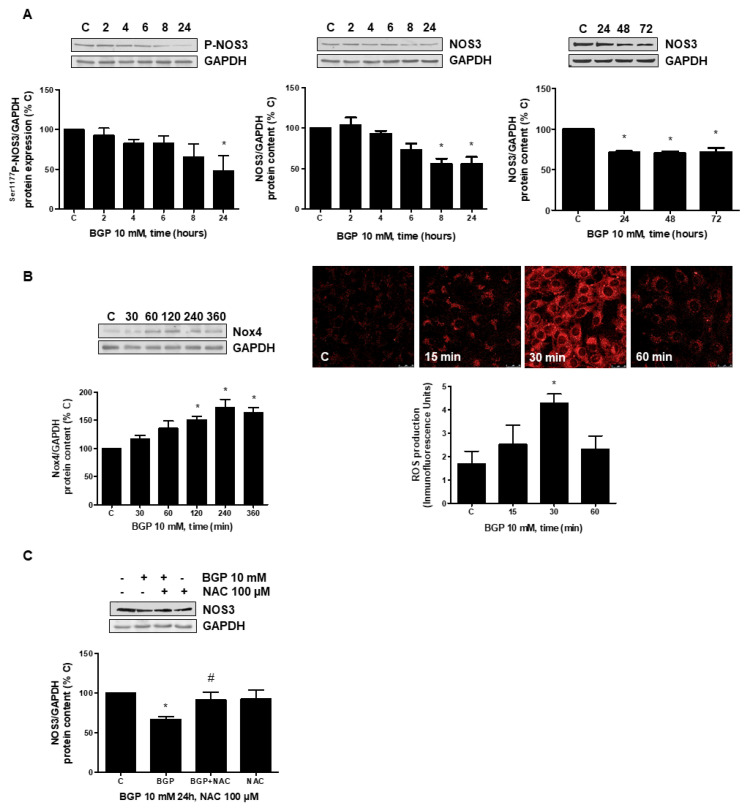
BGP-induced downregulation of NOS3 depends on ROS production in human endothelial cells. A human endothelial cell line (EA.hy926) was treated with 10 mM BGP at different time points (**A**,**B**). Some cells were incubated in the presence of 100 µM N-acetylcysteine (NAC), and then BGP was added for 24 h (**C**). (**A**) NOS3 activity and NOS3 expression were studied by the expression of ^Ser1177^ P-NOS3 and NOS3, respectively, in human endothelial cells treated with 10 mM BGP at different times. (**B**) Oxidative stress was analyzed by Nox4 expression by Western blot (on the left panel) and by ROS production using a CellROX probe that was added during the last 30 min of incubation. ROS production in red fluorescence were visualized by confocal microscopy, representative microphotographs are shown on the right panel with 40× magnification, scale bar and 50 µm, and the densitometric analysis is shown below. (**C**) Endothelial cells were preincubated with 100 µM NAC before adding BGP for 24 h, and then NOS3 expression was evaluated by Western blot. A representative Western blot is included at the top of each panel with the densitometric analysis below. Values are the mean ± SEM of 5 (**A**,**B**) or 4 (**C**) independent experiments, * *p* < 0.05 vs. control cells (C) and # *p* < 0.05 vs. BGP alone.

In order to explore a possible mechanism involved in the reduction in NOS3 expression induced by hyperphosphatemia, in vitro studies were performed in human endothelial cells, analyzing the role of oxidative stress in this effect. Firstly, NOS3 activity and NOS3 expression was assessed at different times in human endothelial cells treated with BGP, an extracellular donor of phosphate. BGP induced a significant reduction in phosphorylation of ^Ser1177^ P-NOS3 protein expression as well as a reduction in NOS3 protein expression, which remained inhibited even until 72 h (Figure 5A), indicating that NOS3 was less effective. Secondly, Nox4 expression was evaluated by Western blot and ROS production by confocal microscopy. BGP induced not only Nox4 expression but also ROS production (Figure 5B). The presence of the antioxidant NAC, a precursor of glutathione, avoided the reduction in NOS3 expression (Figure 5C), suggesting that ROSs are mediating hyperphosphatemia-induced NOS3 downregulation.

**Figure 6:**
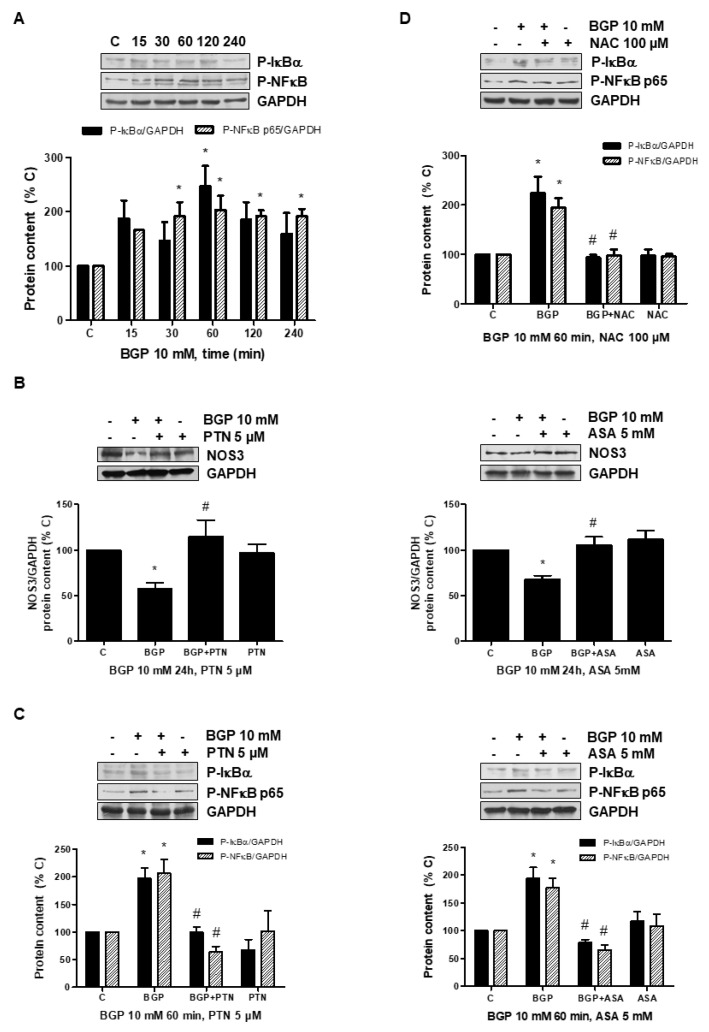
BGP-induced NOS3 downregulation depends on oxidative stress-induced NFκB activation in human endothelial cells. A human endothelial cell line (EA.hy926) was treated with 10 mM BGP. (**A**) Phosphorylation of IκB and NFκB p65 was studied by expression of ^Ser32^ P-IκB and ^Ser536^ P-NFκB p65, respectively, in human endothelial cells treated with 10 mM BGP at different times. (**B**) Endothelial cells were preincubated with 5 µM parthenolide (PTN) or 5 mM acetyl salycilic acid (ASA) before adding BGP for 24 h, and then NOS3 expression was evaluated by Western blot. (**C**,**D**) Endothelial cells were preincubated with 5 µM PTN or 5 mM ASA (panel **C**) and with 100 µM N-acetylcysteine (NAC) (panel **D**) before adding BGP for 60 min, and then ^Ser32^ P-IκB and ^Ser536^ P-NFκB p65 expressions were evaluated by Western blot. A representative Western blot is included at the top of each panel with the densitometric analysis below. Values are the mean ± SEM of 3 independent experiments, * *p* < 0.05 vs. control cells (C) and # *p* < 0.05 vs. BGP alone.

The downregulation of NOS3 through activation of the NFκB transcription factor by different pathways has been described [53,54,55]. For that reason, we explored whether BGP could stimulate NFκB in endothelial cells. BGP induced phosphorylation of both, IκB and NFκB p65, suggesting the activation of NFκB transcription factor (Figure 6A). After that, we analyzed NOS3 expression in cells treated with BGP in the presence of two described inhibitors of NFκB such as parthenolide (PTN) and acetyl salycilic acid (ASA) [56,57]. PTN and ASA blocked significantly BGP effect on NOS3 expression (Figure 6B). Moreover, both inhibited BGP-induced NFκB activation (Figure 6C), suggesting that NFκB are involved in the regulation of NOS3 induced by BGP. Lastly, we explored the activation of NFκB in cells treated with BGP in the presence of NAC in order to assess whether oxidative stress mediates NFκB activation. Figure 6D shows NAC blocked BGP-induced NFκB activation. These results suggest that hyperphosphatemia reduces NOS3 expression through activation of oxidative stress-induced NFκB.

### 3.4. Hyperphosphatemia Induced Inflammation by Increasing Oxidative Stress in Endothelial Cells

**Figure 7:**
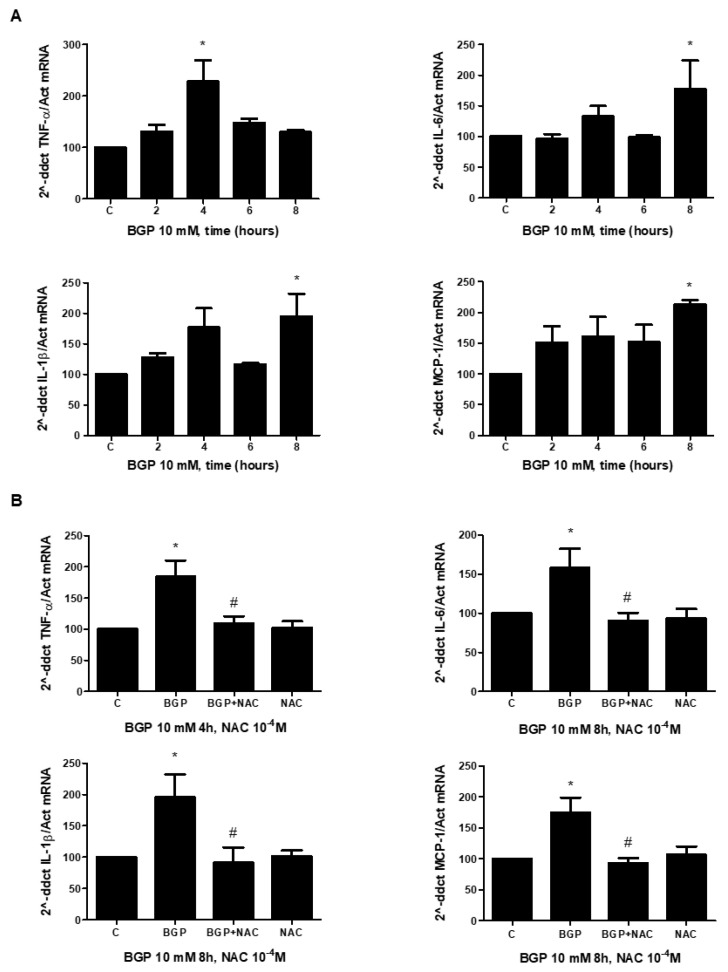
BGP-induced inflammation depends on oxidative stress in human endothelial cells. A human endothelial cell line (EA.hy926) was treated with 10 mM BGP at different times (**A**). Some cells were incubated in the presence of 100 µM N-acetylcysteine (NAC) and then BGP was added at indicated times (**B**). (**A**,**B**) The expressions of several cytokines, TNF-α, IL-6, IL-1ß and MCP-1, were analyzed by real-time PCR in endothelial cells treated with BGP at different times (**A**) or in cells treated with BGP in the presence or the absence of NAC at indicated times (**B**). Values are the mean ± SEM of 6 (**A**) or 4 (**B**) independent experiments, * *p* < 0.05 vs. control cells (C) and # *p* < 0.05 vs. BGP alone.

In order to study a possible mechanism involved in hyperphosphatemia-induced inflammation, in vitro studies were performed in human endothelial cells. The effect of BGP on pro-inflammatory cytokine expression was evaluated in endothelial cells by real time PCR. BGP significantly stimulated the expression of several cytokines, including TNF-alpha, IL-6, IL-1-beta and MCP-1 (Figure 7A). TNF-alpha reached maximum expression levels at 4 h, while the other cytokines required 8 h to reach peak expression levels. As oxidative stress can regulate the inflammation process, endothelial cells were pre-treated with NAC before adding BGP to evaluate cytokine expression. NAC blocked BGP effect on all cytokine expression (Figure 7B), suggesting that oxidative stress could also mediate inflammation.

### 3.5. Hyperphosphatemia Induced Vascular Fibrosis by Increasing Oxidative Stress in Vascular Smooth Muscle Cells

**Figure 8:**
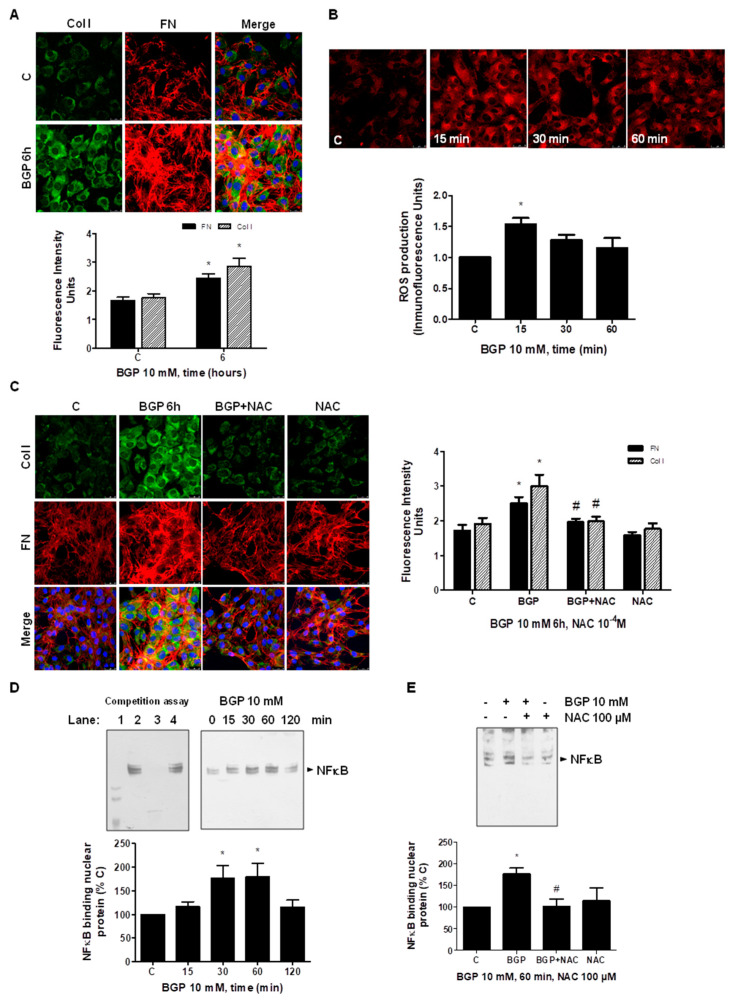
BGP induces fibrosis mediated through ROS-induced NFκB activation in vascular smooth muscle cells. Smooth muscle cells (SMCs) were incubated with 10 mM BGP for 6 h (panels **A**,**C**) or at different times (panels **B**,**D**). (**A**,**C**) Intracellular expressions of Collagen I (Col I, in green and stripped bars) and Fibronectin (FN, in red and closed bars) were assessed by immunofluorescence after treating SMC with BGP for 6 h (panel **A**) or in the presence or absence of 100 µM NAC before adding BGP 6 h (panel **C**). Representative microphotographs are shown with 40× magnification, scale bar and 50 µm. The densitometric analysis is shown next to pictures. (**B**) CellROX probe was added during the last 30 min of incubation. After being washed twice, in vivo cells were visualized by confocal microscopy to test ROS production in red fluorescence. Representative microphotographs are shown with 40× magnification, scale bar, 50 µm. The densitometric analyses are shown below them. (**D**,**E**) Nuclear extracts were harvested from cells incubated with BGP at different times (panel **D**) or in the presence of 100 µM NAC for 60 min (panel **E**). Then, nuclear extracts were incubated with biotin-labeled oligonucleotide containing the NFκB consensus sequence from human FN promoter. (**D**) A representative EMSA is shown join to a competition assay using nuclear extracts from control cells; lane 1, negative control without nuclear extract; lane 2, biotin-labeled NFκB; lane 3, an excess of unlabeled NFκB; lane 4, an excess of unlabeled AP-1. The densitometric analysis is given below the EMSA assay. (**E**) A representative EMSA of cells treated with BGP and NAC is shown with its densitometric analysis below. Values are the mean ± SEM of 6 (**A**–**C**) or 4 (**D**,**E**) independent experiments, * *p* < 0.05 vs. control cells (C) and # *p* < 0.05 vs. BGP alone.

In order to study a possible mechanism involved in hyperphosphatemia-induced vascular fibrosis, in vitro studies were carried out using human SMC, evaluating the role of oxidative stress on vascular fibrosis. The effect of hyperphosphatemia was checked using 10 mM BGP at different times in cultured SMC. The ECM protein expressions, FN and collagen I, were evaluated by immunofluorescence (Figure 8A) and by Western blot (Supplemental Figure S2A). BGP induced the expression of all these proteins, reaching a maximum effect between 6 and 8 h. Furthermore, CTGF expression was analyzed by Western blot, and it increased in the presence of BGP (Supplemental Figure S2A). Then, ROS production was measured by confocal microscopy. BGP significantly increased reactive oxygen species production approximately 15 min after exposure in SMC (Figure 8B). After that, experiments in SMC with BGP in the presence or the absence of the antioxidant NAC to block synthesis of reactive oxygen species were performed to evaluate CTGF, FN or collagen I expressions. NAC significantly blocked the BGP inducer effect on these proteins (Figure 8C and Supplemental Figure S2B). The effect of BGP on fibrosis was specific of phosphate, as it is blocked in the presence of PFA, an antagonist of phosphate transporter Pit-1 (Supplemental Figure S2C). In addition, the role of NFκB factor was studied, as it can regulate the FN promoter. BGP induced the binding of NFκB to nuclear proteins, a biotin-labeled oligo which recognizes specifically the sequence of NFκB transcription factor in human FN promoter (Figure 8D). The effect was maximal between 30 and 60 min after adding BGP. In addition, the presence of NAC inhibited the binding of NFκB as analyzed by EMSA assays (Figure 8E). This result suggests that BGP regulates FN expression through activation of oxidative stress-induced NFκB.

## 4. Discussion

In this work, we demonstrate that hyperphosphatemia associated with age can promote vascular dysfunction by reducing endothelium dependent relaxation and increasing inflammation and vascular fibrosis. We found an important role for oxidant stress in these effects since hyperphosphatemia induces an imbalance between oxidants production and antioxidant capacity. In vivo studies were carried out in a model of aged mice to assess the relaxation, inflammation, fibrosis and the oxidation/antioxidation balance in the aorta. All these parameters were impaired in old mice with respect to young mice, whereas they were improved in old mice fed with a low P diet, suggesting that hyperphosphatemia can be in part responsible for the observed changes. Old mice show hyperphosphatemia possibly because of an alteration of phosphate homeostasis with a decreased Klotho expression and the increment in the Na-Pi cotransporter in the kidney, as we described in previous studies [58].

Endothelial dysfunction is usually related to an imbalance of endothelial vasoactive factors, including a rise of ET-1 and a reduction in NO availability. Several experimental studies in animals and humans have confirmed that the age-related reduction in endothelium-dependent vasodilatation is due to the reduced NO bioavailability caused by a reduced expression in NOS3 [59,60,61,62,63]. In agreement with that, our old mice also exhibit less vascular relaxation than young mice where hyperphosphatemia is potentially responsible, as mice fed with a low P diet significantly improved endothelium-dependent relaxation. Moreover, the effect of L-NAME in the experiments of vascular reactivity support the implication of NOS3. However, the precise mechanism remains unclear. Several alterations involved in the reduction in NOS3 expression as well as NO levels in aged endothelial cells have been studied [63,64,65,66], indicating a relevant role for oxidative stress as antioxidants were able to improve NO bioavailability and endothelial function in older humans and animals. In this sense, we also observed a reduction in NOS3 expression in the aorta from old mice, which improved with low P diet. This association was supported by the significant negative correlation that exists between phosphate serum levels and mRNA NOS3 expression in aorta. On the other side, ROS production was increased in aorta from old mice, and mice fed with low P diet show reduced ROS levels. Similar results were found in vitro using endothelial cells treated with BGP; we found a significant reduction in NOS3 expression and a rise of ROS production. The presence of the antioxidant NAC recovered the reduction in NOS3 induced by BGP. Some authors have described that NFκB-activating stimuli, such as lipopolysaccharide, TNF-α and interleukin-1ß [53,54,55], suppressed eNOS mRNA and protein levels. Consistent with these data, we found in cultured endothelial cells that BGP induced downregulation of NOS3 through the activation of NFκB, which acts as a negative regulator of NOS3 expression via ROS production. This was confirmed with two known inhibitors of NFκB, acetyl salycilic acid [56,57] and parthenolide, as well as with the antioxidant NAC. All of them were able to block BGP-induced NFκB activation. In summary, aging-related hyperphosphatemia seems to be associated with age-related changes in vascular relaxation by NOS3 reduction via ROS-induced NFκB activation.

Aging is also related to inflammation. Thus, the expressions of two typical pro-inflammatory cytokines, IL-6 and MCP-1 were assessed in the aorta. Old mice expressed high levels of IL-6 and MCP-1 with respect to young mice. Restriction in phosphate reduced cytokines levels. IL-6 has a strong chronic inflammatory component [67], it is arguably the most important cytokine across age-related pathologies that is used as a common marker of inflammatory status [47]. Most cytokines interact with cell surface receptors to initiate intracellular signaling cascades that ultimately activate transcription. Among the transcription factors that regulate chronic inflammation across multiple diseases are the NFκB and STAT proteins [68]. Pro-inflammatory condition depends on NFκB signaling in endothelial dysfunction. NFκB proteins are sequestered in the cytoplasm by binding to IκB proteins; phosphorylation of IκB in response to inflammatory stimuli or oxidative stress results in its degradation and enables nuclear translocation of NFκB where it can activate gene transcription of pro-inflammatory cytokines. To study whether ROS can be implicated in the induction of pro-inflammatory cytokines, endothelial cells were treated with BGP. BGP significantly induced the expression of different cytokines apart from ROS production. When cells were preincubated with NAC, all cytokine expression was reduced in a significant way. Therefore, aging-related hyperphosphatemia induced vascular inflammation through ROS production. This effect could probably also be mediated by the activation of NFκB, similar to the situation with FN, but more studies are required to definitively prove this.

Aging is generally characterized by increased fibrotic tissue deposition in many organs [69]. Vascular fibrosis was assessed in the aorta as another factor involved in vascular dysfunction. Aorta from old mice showed more expression of FN and collagen than young mice; the Sirius red/elastin ratio was completely inverted, making the arterial wall more stiff and favoring vascular dysfunction. Small vessels of other tissues such as heart and kidney also demonstrated more fibrosis in old mice. Phosphate dietary restriction reduced vascular fibrosis in all tissues studied. As the appearance of fibrosis in aorta was mainly located in the media layer of SMC, the mechanisms involved in hyperphosphatemia-induced fibrosis were studied in vitro in SMC treated with BGP. Many studies have demonstrated the association of fibrosis and increased oxidative stress in the pathogenesis of some chronic human diseases [70,71,72]. Oxidative stress was increased not only in aorta from old mice but also in SMC treated with BGP. Our data suggest that fibrosis in SMC was mediated by ROS production, as it was blocked in the presence of the antioxidant NAC.

Adler et al. found that the transcription factor most strongly associated with aging was NFκB [73], which can drive several aging phenotypes in the skin, spine and brain [73,74,75]. Wu J et al. [76], reviewed the role of oxidative stress, inflammation and fibrosis in cardiovascular aging, suggesting the involvement of two important factors, NFκB and Nrf2, among others. It is well known that NFκB can be activated by oxidative stress in many cell types [77] to later induce transcription of many genes by binding to specific target sites in their promoter regions, for instance, human FN [52]. The role of NFκB factor regulating FN expression was studied in SMC treated with BGP. BGP induced the binding of NFκB to the FN promoter, and the presence of NAC blocked that effect, suggesting that ROS are involved in the BGP-induced FN expression through NFκB activation.

Finally, as aging is also related to oxidative stress, the oxidant/antioxidant balance was assessed in aorta. NADPH (nicotinamide adenine dinucleotide phosphate) oxidase is an important source of reactive oxygen species. In vascular cells, several isoforms of NADPH oxidases (Nox1, Nox2, Nox4 and Nox5) are expressed [78]; thus, we explored Nox4 expression that could mediate ROS production. Higher expression of Nox4 was found in old mice than in young mice, and the low P diet reduced those levels. Similar results were found in vitro by using endothelial cells treated with BGP. Antioxidant enzymes are mainly regulated by Nrf2 factor, which can stimulate the expression of superoxide dismutase or glutathione peroxidase between others [34,79]. Hecker et al. [34] suggested that loss of cellular redox homeostasis promotes profibrotic myofibroblast phenotypes that result in persistent fibrosis associated with aging, finding a Nox4/Nrf2 imbalance. According to that, we also found a Nox4/Nrf2 imbalance in the aorta from old mice with respect to young mice. Moreover, Sod2-Mn and Gpx-1 were also downregulated probably as a consequence of a reduction in Nrf2 factor expression. However, low P diet did not modify Nrf2 and Sod nor Gpx expressions, which still continued low. This fact can be explained because Nrf2 is reduced with aging [34], and it is probably not regulated by phosphate; however, we have not assessed the BGP effect on Nrf2 regulation in vitro, so further studies would be need to elucidate this point. Our data suggest aging-related oxidative stress is the result from an elevated expression of the reactive oxygen species-generating enzyme, Nox4, and an impaired capacity to induce the Nrf2 factor antioxidant response.

Apart from loss of redox homeostasis, aortas from old mice also presented higher expression of NOS2 that was accompanied with more nitrotyrosine expression. These results suggest that NO synthetized from NOS2 provoked nitrosative damage. It is known that nitrosative stress is a major factor responsible for endothelial dysfunction, especially through peroxynitrite that is a product result of reacting NO with ROS. Peroxynitrite could be desacoplating NOS3, reducing its activity as previously described [80]; however, the measure of peroxinitrite has not been addressed in this study. A limitation of this study is the small size of the aorta that it is insufficient for performing more assays such as the measure of NOS3 activity or peroxinitrite.

In summary, these data suggest that aging-related vascular dysfunction depends, at least partially, on hyperphosphatemia associated with advanced age in mice. We propose here that hyperphosphatemia induces an imbalance oxidant/antioxidant in favour of oxidative stress, which on one hand reduces endothelium-dependent relaxation through NOS3 reduction and on the other hand increases inflammation and the fibrosis increasing vascular stiffness. The proposed mechanism is that BGP could activate the NFκB transcription factor by increasing ROS production to regulate both the downregulation of NOS3 expression in EC and the induction of FN expression in SMC. However, the involvement of NFκB in BGP-induced inflammation would need further studies. The relevance of finding different dietary supplements that can improve endothelial dysfunction should be investigated in the near future.

## Data Availability

Not applicable.

## Supplementary Materials

The following are available online at https://www.mdpi.com/article/10.3390/antiox10081308/s1, Figure S1. Additional data from in vivo studies in old mice. Male C57Bl6 mice from 5 month old (Young, closed bars), 24 month old fed with normal diet (Old-24 m, striped bars) and 24 month-old mice fed with a low phosphate diet for the last 3 months (Old-24 m Low P, grey bars) were used. (A) General data from all groups of mice studied (10 mice per group) are shown in a table including body weight and BMI, as well as water and food intake per day. (B) Graphs of correlations between the mRNA expression of pro-inflammatory cytokine IL-6 with P serum levels and with the fibrosis parameter FN are shown: IL-6 expression and P levels (Pearson r = 0.6789, *p* = 0.0002), IL-6 expression and FN expression (Pearson r = 0.5813, *p* = 0.0036). Values are from 8 mice per group. (C) Sirius red staining of the heart and kidney slices from mice (40×) is shown on the left of panel C with the graph of densitometric analysis on the right. Scale bar, 100 µm. Values are the mean ± SEM of 6 mice per group, * *p* < 0.05 vs. young, # *p* < 0.05 vs. Old-24 m, Figure S2. BGP-induced fibrosis in vascular smooth muscle cells. Smooth muscle cells (SMCs) were treated with 10 mM BGP at indicated times. (A) CTGF, Collagen I (Col I) and Fibronectin (FN) were assessed by Western blot. (B) SMCs were incubated with 10 mM BGP for 6 h in the presence or absence of 100 µM NAC to assay CTGF and FN expression by Western blot. (C) To study the specific effect of BGP, cells were pre-incubated with 1 mM PFA, an antagonist of cotransporter Na-P termed Pit-1, and then FN protein content was assessed by Western blot (on the left panel C), and FN and Col I expression were analyzed by immunofluorescence (on the right panel (C). Representative microphotographs of immunofluorescence are shown with 40× magnification, scale bar, 50 µm, with their densitometric analyses below pictures. A representative Western blot is included at the top of each panel with the densitometric analysis below. Values are the mean ± SEM of 8 (A) or 4 (B,C) independent experiments, * *p* < 0.05 vs. control cells (C) and # *p* < 0.05 vs. BGP alone.
