# Supplementary material for: Hyperphosphatemia-Induced Oxidant/Antioxidant Imbalance Impairs Vascular Relaxation and Induces Inflammation and Fibrosis in Old Mice
Source: Antioxidants (Basel). 2021 Aug 19;10(8):1308. doi: 10.3390/antiox10081308 (PMC8389342; doi:10.3390/antiox10081308)
Supplement: Supplementary file 1 [file antioxidants-10-01308-s001.zip › antioxidants-1331784-supplementary.pdf]

A

General data of mice	Young	Old-24m	Old-24m Low P
Body weight (g)	31.51 ± 0.72	36.03 ± 1.61	34.58 ± 1.52
BMI (Kg/m²)	3.51 ± 0.07	3.47 ± 0.13	3.23 ± 0.08
Water intake (mL/animal/day)	4.08 ± 10.15	3.96 ± 0.14	3.86 ± 0.11
Food intake (g/animal/day)	3.36 ± 0.15	3.62 ± 0.21	3.55 ± 0.20

B

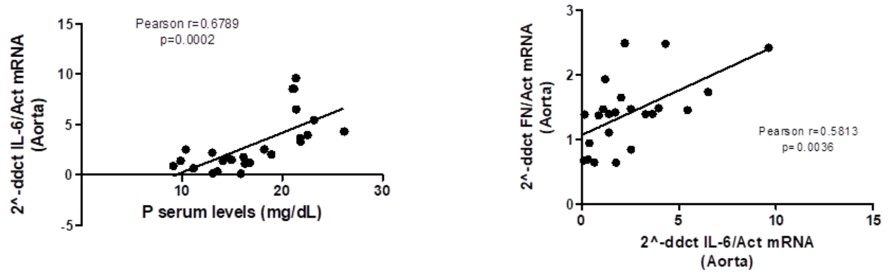

C

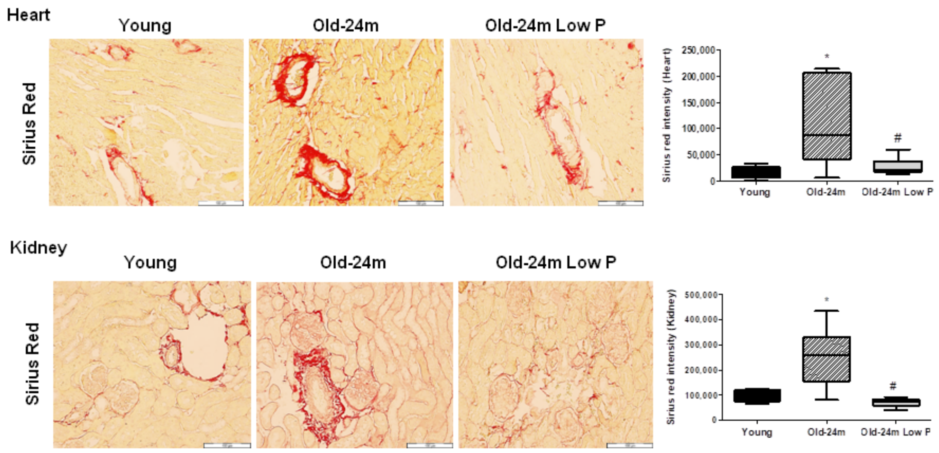

Figure S1. Additional data from in vivo studies in old mice. Male C57Bl6 mice from 5 month old (Young, closed bars), 24 month old fed with normal diet (Old-24 m, striped bars) and 24 month-old mice fed with a low phosphate diet for the last 3 months (Old-24 m Low P, grey bars) were used. (A) General data from all groups of mice studied (10 mice per group) are shown in a table including body weight and BMI, as well as water and food intake per day. (B) Graphs of correlations between the mRNA expression of pro-inflammatory cytokine IL-6 with P serum levels and with the fibrosis parameter FN are shown: IL-6 expression and P levels (Pearson r = 0.6789, p = 0.0002), IL-6 expression and FN expression (Pearson r = 0.5813, p = 0.0036). Values are from 8 mice per group. (C) Sirius red staining of the heart and kidney slices from mice (40x) is shown on the left of panel C with the graph of densitometric analysis on the right. Scale bar, 100 µm. Values are the mean ± SEM of 6 mice per group, * $p < 0.05$ vs. young, # $p < 0.05$ vs. Old-24 m.

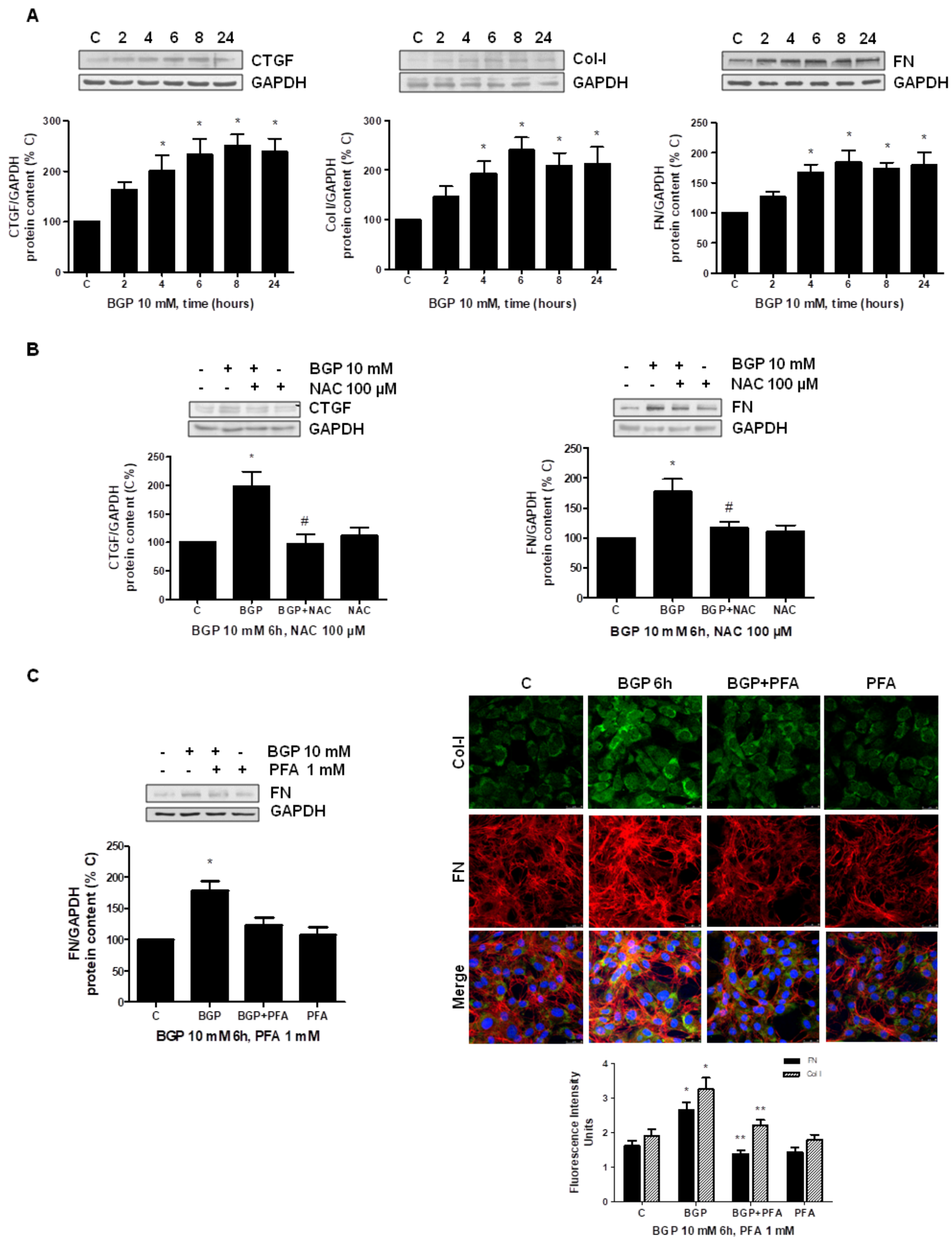

Figure S2. BGP-induced fibrosis in vascular smooth muscle cells. Smooth muscle cells (SMCs) were treated with 10 mM BGP at indicated times. (A) CTGF, Collagen I (Col I) and Fibronectin (FN) were assessed by Western blot. (B) SMCs were incubated with 10 mM BGP for 6 h in the presence or absence of 100 µM NAC to assay CTGF and FN expression by Western blot. (C) To study the specific effect of BGP, cells were pre-incubated with 1 mM PFA, an antagonist of cotransporter Na-P termed Pit-1, and then FN protein content was assessed by Western blot (on the left panel C), and FN and Col I expression were analyzed by immunofluorescence (on the right panel (C). Representative microphotographs of immunofluorescence are shown with 40 x magnification, scale bar, 50 µm, with their densitometric analyses below pictures. A representative Western blot is included at the top of each panel with the densitometric analysis below. Values are the mean ± SEM of 8 (A) or 4 (B,C) independent experiments, * $p < 0.05$ vs. control cells (C) and # $p < 0.05$ vs. BGP alone.
